# 1-(4-Chloro­phen­yl)-2-phenyl-2-(3-phenyl-1-isoquinolylsulfan­yl)ethanone

**DOI:** 10.1107/S1600536809041282

**Published:** 2009-10-17

**Authors:** F. Nawaz Khan, P. Manivel, K. Prabakaran, Venkatesha R. Hathwar, Seik Weng Ng

**Affiliations:** a Chemistry Division, School of Science and Humanities, VIT University, Vellore 632 014, Tamil Nadu, India; b Solid State and Structural Chemistry Unit, Indian Institute of Science, Bangalore 560 012, Karnataka, India; c Department of Chemistry, University of Malaya, 50603 Kuala Lumpur, Malaysia

## Abstract

The title compound, C_29_H_20_ClNOS, is a 1-substituted-3-phenyl­isoquinoline that crystallizes with four independent mol­ecules in the asymmtric unit. The four mol­ecules have similar C—S—C angles. The most noteworthy differences between the mol­ecules relate to the inclination of the 3-phenyl subsituent with respect to the isoquinoline fused-ring [dihedral angles of 21.2 (1), 25.6 (2), 34.3 (1) and 36.5 (2)°].

## Related literature

For the crystal structure of 1-(4-chloro-3-fluoro­phen­yl)-2-[(3-phenyl­isoquinolin-1-yl)sulfan­yl]ethanone, see: Manivel *et al.* (2009 ▶).
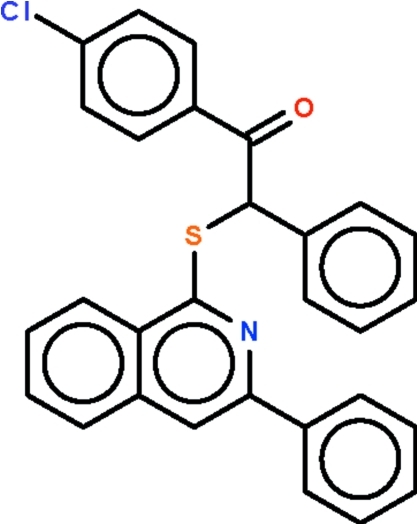


## Experimental

### 

#### Crystal data

C_29_H_20_ClNOS
*M* *_r_* = 465.97
Triclinic, 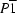

*a* = 10.2808 (6) Å
*b* = 11.1145 (7) Å
*c* = 42.169 (3) Å
α = 97.562 (1)°
β = 96.647 (2)°
γ = 90.043 (1)°
*V* = 4743.9 (5) Å^3^
*Z* = 8
Mo *K*α radiation
μ = 0.27 mm^−1^
*T* = 290 K
0.30 × 0.24 × 0.17 mm

#### Data collection

Bruker SMART area-detector diffractometer
Absorption correction: multi-scan (*SADABS*; Sheldrick, 1996 ▶) *T*_min_ = 0.923, *T*_max_ = 0.955
45825 measured reflections
16659 independent reflections
10228 reflections with *I* > 2σ(*I*)
*R*_int_ = 0.048

#### Refinement

*R*[*F* ^2^ > 2σ(*F* ^2^)] = 0.079
*wR*(*F* ^2^) = 0.170
*S* = 1.18
16659 reflections
1189 parameters
H-atom parameters constrained
Δρ_max_ = 0.20 e Å^−3^
Δρ_min_ = −0.20 e Å^−3^

### 

Data collection: *SMART* (Bruker, 2004 ▶); cell refinement: *SAINT* (Bruker, 2004 ▶); data reduction: *SAINT*; program(s) used to solve structure: *SHELXS97* (Sheldrick, 2008 ▶); program(s) used to refine structure: *SHELXL97* (Sheldrick, 2008 ▶); molecular graphics: *X-SEED* (Barbour, 2001 ▶); software used to prepare material for publication: *publCIF* (Westrip, 2009 ▶).

## Supplementary Material

Crystal structure: contains datablocks global, I. DOI: 10.1107/S1600536809041282/tk2556sup1.cif

Structure factors: contains datablocks I. DOI: 10.1107/S1600536809041282/tk2556Isup2.hkl

Additional supplementary materials:  crystallographic information; 3D view; checkCIF report

## Figures and Tables

**Fig. 1.**
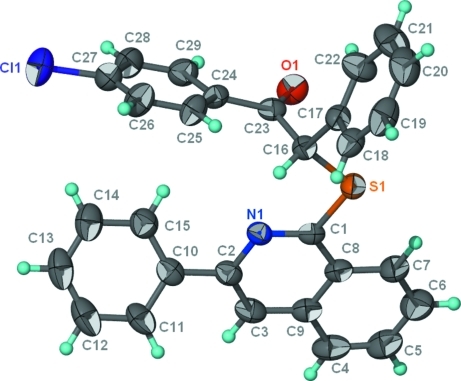
Thermal ellipsoid plot (Barbour, 2001) of one of the four independent molecules of C29H20NOS at the 50% probability level; hydrogen atoms are drawn as spheres of arbitrary radius.

## supplementary crystallographic information

### Experimental

1-Mercapto-3-phenylisoquinoline (10.0 mmol) and 2-bromo-1-(4-chlorophenyl)-2-phenylethanone (10.5 mmol) were heated in ethanol (50 ml) under a nitrogen atmosphere for 2 h. The solid product was collected and dissolved in chloroform. The chloroform solution was washed with water and dried; the dry solution was concentrated. The solid material was purified by recrystallization from ether.

### Refinement

Carbon-bound H-atoms were placed in calculated positions (C–H 0.93–0.98 Å) and were included in the refinement in the riding model approximation with *U*_iso_(H) set to 1.2*U*_eq_(C).

### Crystal data

**Table:**

C_29_H_20_ClNOS	*Z* = 8
*M**_r_* = 465.97	*F*(000) = 1936
Triclinic, *P*1	*D*_x_ = 1.305 Mg m^−^^3^
Hall symbol: -P 1	Mo *K*α radiation, λ = 0.71073 Å
*a* = 10.2808 (6) Å	Cell parameters from 1324 reflections
*b* = 11.1145 (7) Å	θ = 1.6–24.3°
*c* = 42.169 (3) Å	µ = 0.27 mm^−^^1^
α = 97.562 (1)°	*T* = 290 K
β = 96.647 (2)°	Block, colorless
γ = 90.043 (1)°	0.30 × 0.24 × 0.17 mm
*V* = 4743.9 (5) Å^3^	

### Data collection

**Table:**

Bruker SMART area-detector diffractometer	16659 independent reflections
Radiation source: fine-focus sealed tube	10228 reflections with *I* > 2σ(*I*)
graphite	*R*_int_ = 0.048
φ and ω scans	θ_max_ = 25.0°, θ_min_ = 1.5°
Absorption correction: multi-scan (*SADABS*; Sheldrick, 1996)	*h* = −12→12
*T*_min_ = 0.923, *T*_max_ = 0.955	*k* = −13→13
45825 measured reflections	*l* = −50→50

### Refinement

**Table:**

Refinement on *F*^2^	Primary atom site location: structure-invariant direct methods
Least-squares matrix: full	Secondary atom site location: difference Fourier map
*R*[*F*^2^ > 2σ(*F*^2^)] = 0.079	Hydrogen site location: inferred from neighbouring sites
*wR*(*F*^2^) = 0.170	H-atom parameters constrained
*S* = 1.18	*w* = 1/[σ^2^(*F*_o_^2^) + (0.0369*P*)^2^ + 2.6389*P*] where *P* = (*F*_o_^2^ + 2*F*_c_^2^)/3
16659 reflections	(Δ/σ)_max_ = 0.001
1189 parameters	Δρ_max_ = 0.20 e Å^−^^3^
0 restraints	Δρ_min_ = −0.20 e Å^−^^3^

### Fractional atomic coordinates and isotropic or equivalent isotropic displacement parameters (Å^2^)

**Table:**

	*x*	*y*	*z*	*U*_iso_*/*U*_eq_	
S1	0.42109 (11)	0.61616 (11)	0.07142 (3)	0.0553 (3)	
S2	0.14531 (11)	0.11317 (11)	0.06884 (3)	0.0579 (3)	
S3	0.07836 (11)	0.54707 (11)	0.42913 (3)	0.0567 (3)	
S4	0.35545 (11)	0.04298 (11)	0.43006 (3)	0.0585 (3)	
Cl1	0.10000 (18)	1.01418 (16)	0.22600 (4)	0.1147 (6)	
Cl2	0.61254 (15)	0.50839 (14)	0.22440 (3)	0.0899 (5)	
Cl3	0.39882 (16)	0.78415 (17)	0.27399 (4)	0.1059 (6)	
Cl4	−0.10384 (17)	0.28896 (15)	0.27441 (4)	0.1043 (5)	
O1	0.2278 (3)	0.5609 (3)	0.11499 (8)	0.0651 (9)	
O2	0.3808 (3)	0.0567 (3)	0.11194 (8)	0.0649 (9)	
O3	0.2759 (3)	0.4471 (3)	0.38692 (7)	0.0641 (9)	
O4	0.1214 (3)	−0.0536 (3)	0.38623 (8)	0.0676 (9)	
N1	0.5469 (3)	0.7207 (3)	0.12701 (8)	0.0449 (8)	
N2	0.0699 (3)	0.2136 (3)	0.12441 (9)	0.0497 (9)	
N3	−0.0485 (3)	0.5935 (3)	0.37340 (8)	0.0443 (8)	
N4	0.4325 (3)	0.0908 (3)	0.37470 (8)	0.0488 (9)	
C1	0.5563 (4)	0.6405 (4)	0.10171 (10)	0.0442 (10)	
C2	0.6565 (4)	0.7432 (4)	0.14938 (11)	0.0513 (11)	
C3	0.7697 (4)	0.6814 (4)	0.14609 (12)	0.0599 (13)	
H3	0.8414	0.6982	0.1617	0.072*	
C4	0.8947 (5)	0.5242 (5)	0.11514 (15)	0.0709 (15)	
H4	0.9686	0.5384	0.1301	0.085*	
C5	0.8961 (5)	0.4388 (5)	0.08922 (15)	0.0751 (15)	
H5	0.9715	0.3940	0.0868	0.090*	
C6	0.7863 (5)	0.4157 (5)	0.06576 (15)	0.0820 (16)	
H6	0.7897	0.3571	0.0480	0.098*	
C7	0.6758 (5)	0.4798 (4)	0.06949 (12)	0.0651 (13)	
H7	0.6030	0.4643	0.0542	0.078*	
C8	0.6694 (4)	0.5695 (4)	0.09612 (11)	0.0503 (11)	
C9	0.7796 (4)	0.5918 (4)	0.11926 (12)	0.0535 (12)	
C10	0.6416 (5)	0.8365 (4)	0.17693 (11)	0.0534 (12)	
C11	0.7456 (5)	0.9117 (5)	0.19087 (12)	0.0718 (15)	
H11	0.8258	0.9046	0.1827	0.086*	
C12	0.7303 (7)	0.9976 (5)	0.21713 (14)	0.0850 (18)	
H12	0.8012	1.0469	0.2266	0.102*	
C13	0.6139 (7)	1.0107 (5)	0.22922 (12)	0.0787 (17)	
H13	0.6056	1.0683	0.2470	0.094*	
C14	0.5096 (6)	0.9402 (5)	0.21551 (12)	0.0765 (16)	
H14	0.4295	0.9497	0.2237	0.092*	
C15	0.5225 (5)	0.8539 (4)	0.18924 (11)	0.0645 (13)	
H15	0.4501	0.8067	0.1797	0.077*	
C16	0.3010 (4)	0.7185 (4)	0.08743 (10)	0.0469 (11)	
H16	0.3427	0.7977	0.0954	0.056*	
C17	0.1996 (4)	0.7312 (4)	0.05883 (10)	0.0496 (11)	
C18	0.2187 (5)	0.8173 (4)	0.03890 (11)	0.0610 (13)	
H18	0.2907	0.8699	0.0437	0.073*	
C19	0.1304 (6)	0.8252 (5)	0.01176 (12)	0.0798 (17)	
H19	0.1432	0.8839	−0.0014	0.096*	
C20	0.0249 (7)	0.7478 (7)	0.00426 (14)	0.0926 (19)	
H20	−0.0326	0.7525	−0.0143	0.111*	
C21	0.0041 (6)	0.6640 (6)	0.02393 (15)	0.0936 (19)	
H21	−0.0691	0.6129	0.0193	0.112*	
C22	0.0919 (5)	0.6549 (5)	0.05077 (13)	0.0753 (15)	
H22	0.0781	0.5957	0.0637	0.090*	
C23	0.2408 (4)	0.6697 (4)	0.11495 (10)	0.0482 (11)	
C24	0.1986 (4)	0.7581 (4)	0.14110 (10)	0.0457 (10)	
C25	0.1895 (4)	0.8808 (4)	0.13938 (11)	0.0604 (13)	
H25	0.2056	0.9108	0.1206	0.073*	
C26	0.1565 (5)	0.9601 (5)	0.16544 (13)	0.0741 (15)	
H26	0.1500	1.0428	0.1643	0.089*	
C27	0.1340 (5)	0.9144 (5)	0.19270 (13)	0.0713 (15)	
C28	0.1417 (5)	0.7926 (5)	0.19530 (13)	0.0746 (15)	
H28	0.1265	0.7635	0.2143	0.090*	
C29	0.1725 (4)	0.7147 (5)	0.16909 (12)	0.0633 (13)	
H29	0.1759	0.6317	0.1702	0.076*	
C30	0.0383 (4)	0.1341 (4)	0.09883 (10)	0.0474 (11)	
C31	−0.0183 (4)	0.2355 (4)	0.14699 (11)	0.0530 (12)	
C32	−0.1345 (5)	0.1720 (4)	0.14294 (13)	0.0637 (13)	
H32	−0.1921	0.1874	0.1584	0.076*	
C33	−0.2871 (5)	0.0162 (5)	0.11128 (14)	0.0670 (14)	
H33	−0.3471	0.0290	0.1262	0.080*	
C34	−0.3135 (5)	−0.0676 (5)	0.08507 (15)	0.0759 (16)	
H34	−0.3916	−0.1121	0.0823	0.091*	
C35	−0.2260 (5)	−0.0891 (5)	0.06191 (14)	0.0786 (16)	
H35	−0.2461	−0.1472	0.0440	0.094*	
C36	−0.1107 (5)	−0.0242 (4)	0.06574 (13)	0.0694 (14)	
H36	−0.0522	−0.0382	0.0504	0.083*	
C37	−0.0807 (4)	0.0637 (4)	0.09289 (11)	0.0503 (11)	
C38	−0.1695 (4)	0.0842 (4)	0.11611 (12)	0.0556 (12)	
C39	0.0227 (5)	0.3273 (4)	0.17488 (11)	0.0524 (11)	
C40	−0.0677 (5)	0.3925 (5)	0.19212 (13)	0.0769 (15)	
H40	−0.1568	0.3788	0.1859	0.092*	
C41	−0.0271 (6)	0.4763 (5)	0.21811 (14)	0.0840 (17)	
H41	−0.0894	0.5174	0.2295	0.101*	
C42	0.1039 (6)	0.5015 (5)	0.22784 (12)	0.0741 (15)	
H42	0.1307	0.5587	0.2456	0.089*	
C43	0.1937 (5)	0.4391 (5)	0.21048 (12)	0.0710 (14)	
H43	0.2827	0.4541	0.2165	0.085*	
C44	0.1530 (5)	0.3547 (4)	0.18427 (11)	0.0637 (13)	
H44	0.2155	0.3151	0.1726	0.076*	
C45	0.2801 (4)	0.2157 (4)	0.08546 (10)	0.0477 (11)	
H45	0.2453	0.2937	0.0941	0.057*	
C46	0.3547 (4)	0.2334 (4)	0.05736 (10)	0.0482 (11)	
C47	0.3183 (5)	0.3232 (4)	0.03839 (11)	0.0622 (13)	
H47	0.2504	0.3748	0.0435	0.075*	
C48	0.3820 (6)	0.3371 (6)	0.01172 (13)	0.0824 (17)	
H48	0.3573	0.3982	−0.0007	0.099*	
C49	0.4815 (7)	0.2608 (7)	0.00379 (13)	0.091 (2)	
H49	0.5244	0.2700	−0.0140	0.109*	
C50	0.5168 (6)	0.1716 (6)	0.02208 (15)	0.0924 (19)	
H50	0.5837	0.1193	0.0166	0.111*	
C51	0.4549 (5)	0.1579 (5)	0.04858 (13)	0.0778 (16)	
H51	0.4810	0.0967	0.0609	0.093*	
C52	0.3672 (4)	0.1656 (4)	0.11212 (10)	0.0466 (11)	
C53	0.4354 (4)	0.2524 (4)	0.13870 (10)	0.0444 (10)	
C54	0.4428 (4)	0.3758 (4)	0.13719 (11)	0.0572 (12)	
H54	0.4097	0.4065	0.1185	0.069*	
C55	0.4995 (5)	0.4535 (4)	0.16347 (12)	0.0643 (13)	
H55	0.5043	0.5364	0.1623	0.077*	
C56	0.5484 (4)	0.4096 (5)	0.19114 (11)	0.0602 (13)	
C57	0.5453 (5)	0.2867 (5)	0.19298 (11)	0.0633 (13)	
H57	0.5806	0.2564	0.2116	0.076*	
C58	0.4886 (4)	0.2090 (4)	0.16647 (11)	0.0552 (12)	
H58	0.4864	0.1259	0.1675	0.066*	
C59	−0.0568 (4)	0.5391 (4)	0.39884 (11)	0.0469 (11)	
C60	−0.1576 (4)	0.5924 (4)	0.35095 (11)	0.0512 (11)	
C61	−0.2698 (4)	0.5344 (4)	0.35485 (12)	0.0602 (13)	
H61	−0.3419	0.5355	0.3394	0.072*	
C62	−0.3943 (4)	0.4108 (4)	0.38677 (13)	0.0650 (14)	
H62	−0.4691	0.4103	0.3721	0.078*	
C63	−0.3958 (5)	0.3520 (5)	0.41298 (15)	0.0745 (16)	
H63	−0.4718	0.3117	0.4160	0.089*	
C64	−0.2857 (5)	0.3513 (5)	0.43526 (14)	0.0769 (15)	
H64	−0.2878	0.3090	0.4528	0.092*	
C65	−0.1745 (5)	0.4121 (4)	0.43169 (13)	0.0689 (14)	
H65	−0.1017	0.4125	0.4470	0.083*	
C66	−0.1693 (4)	0.4742 (4)	0.40486 (11)	0.0514 (11)	
C67	−0.2806 (4)	0.4724 (4)	0.38164 (12)	0.0534 (12)	
C68	−0.1420 (4)	0.6565 (4)	0.32276 (10)	0.0501 (11)	
C69	−0.2493 (5)	0.6979 (5)	0.30449 (13)	0.0786 (16)	
H69	−0.3329	0.6863	0.3101	0.094*	
C70	−0.2342 (6)	0.7561 (5)	0.27811 (14)	0.0853 (17)	
H70	−0.3077	0.7814	0.2659	0.102*	
C71	−0.1128 (6)	0.7768 (4)	0.26987 (12)	0.0688 (14)	
H71	−0.1026	0.8159	0.2521	0.083*	
C72	−0.0060 (5)	0.7390 (4)	0.28833 (12)	0.0678 (14)	
H72	0.0775	0.7538	0.2831	0.081*	
C73	−0.0196 (5)	0.6797 (4)	0.31429 (11)	0.0584 (12)	
H73	0.0546	0.6547	0.3264	0.070*	
C74	0.2000 (4)	0.6320 (4)	0.41328 (10)	0.0469 (11)	
H74	0.1588	0.7026	0.4049	0.056*	
C75	0.3000 (4)	0.6750 (4)	0.44182 (10)	0.0482 (11)	
C76	0.4083 (5)	0.6089 (5)	0.45012 (13)	0.0773 (16)	
H76	0.4253	0.5377	0.4371	0.093*	
C77	0.4928 (6)	0.6470 (6)	0.47773 (16)	0.0961 (19)	
H77	0.5639	0.5992	0.4833	0.115*	
C78	0.4744 (7)	0.7507 (7)	0.49652 (14)	0.096 (2)	
H78	0.5338	0.7766	0.5145	0.115*	
C79	0.3670 (7)	0.8181 (6)	0.48887 (13)	0.0870 (18)	
H79	0.3519	0.8891	0.5022	0.104*	
C80	0.2798 (5)	0.7818 (4)	0.46142 (11)	0.0625 (13)	
H80	0.2080	0.8294	0.4563	0.075*	
C81	0.2611 (4)	0.5553 (4)	0.38657 (10)	0.0472 (11)	
C82	0.3034 (4)	0.6155 (4)	0.35966 (10)	0.0447 (10)	
C83	0.3152 (4)	0.7404 (4)	0.36117 (11)	0.0596 (12)	
H83	0.3011	0.7899	0.3800	0.071*	
C84	0.3475 (5)	0.7917 (5)	0.33525 (12)	0.0681 (14)	
H84	0.3568	0.8755	0.3366	0.082*	
C85	0.3660 (4)	0.7189 (5)	0.30744 (12)	0.0635 (13)	
C86	0.3579 (5)	0.5957 (5)	0.30527 (12)	0.0692 (14)	
H86	0.3729	0.5471	0.2864	0.083*	
C87	0.3269 (4)	0.5436 (4)	0.33184 (11)	0.0578 (12)	
H87	0.3220	0.4596	0.3307	0.069*	
C88	0.4639 (4)	0.0348 (4)	0.40022 (10)	0.0458 (10)	
C89	0.5224 (4)	0.0906 (4)	0.35278 (11)	0.0526 (11)	
C90	0.6387 (5)	0.0307 (4)	0.35657 (12)	0.0630 (13)	
H90	0.6969	0.0315	0.3413	0.076*	
C91	0.7890 (5)	−0.0957 (5)	0.38808 (14)	0.0703 (14)	
H91	0.8493	−0.0974	0.3732	0.084*	
C92	0.8152 (5)	−0.1544 (5)	0.41401 (16)	0.0795 (16)	
H92	0.8933	−0.1960	0.4168	0.095*	
C93	0.7265 (5)	−0.1536 (5)	0.43686 (15)	0.0801 (16)	
H93	0.7455	−0.1949	0.4546	0.096*	
C94	0.6115 (5)	−0.0917 (4)	0.43297 (12)	0.0674 (14)	
H94	0.5529	−0.0905	0.4482	0.081*	
C95	0.5820 (4)	−0.0305 (4)	0.40621 (11)	0.0514 (11)	
C96	0.6712 (4)	−0.0315 (4)	0.38316 (12)	0.0556 (12)	
C97	0.4834 (5)	0.1576 (4)	0.32549 (11)	0.0552 (12)	
C98	0.5761 (5)	0.2219 (5)	0.31243 (12)	0.0709 (14)	
H98	0.6635	0.2228	0.3211	0.085*	
C99	0.5390 (7)	0.2846 (5)	0.28655 (14)	0.0857 (18)	
H99	0.6015	0.3279	0.2782	0.103*	
C100	0.4091 (7)	0.2830 (5)	0.27303 (13)	0.0848 (18)	
H100	0.3841	0.3231	0.2553	0.102*	
C101	0.3186 (6)	0.2213 (5)	0.28635 (14)	0.0843 (17)	
H101	0.2311	0.2207	0.2777	0.101*	
C102	0.3546 (5)	0.1608 (5)	0.31200 (12)	0.0690 (14)	
H102	0.2906	0.1205	0.3207	0.083*	
C103	0.2225 (4)	0.1309 (4)	0.41367 (10)	0.0470 (11)	
H103	0.2586	0.2010	0.4056	0.056*	
C104	0.1470 (4)	0.1752 (4)	0.44165 (10)	0.0510 (11)	
C105	0.1837 (5)	0.2833 (4)	0.46091 (11)	0.0619 (13)	
H105	0.2514	0.3305	0.4559	0.074*	
C106	0.1194 (6)	0.3217 (6)	0.48775 (13)	0.0834 (17)	
H106	0.1444	0.3945	0.5006	0.100*	
C107	0.0206 (7)	0.2535 (7)	0.49527 (14)	0.097 (2)	
H107	−0.0224	0.2801	0.5131	0.116*	
C108	−0.0157 (6)	0.1463 (6)	0.47688 (16)	0.098 (2)	
H108	−0.0823	0.0990	0.4823	0.118*	
C109	0.0469 (5)	0.1076 (5)	0.45008 (13)	0.0753 (15)	
H109	0.0210	0.0346	0.4375	0.090*	
C110	0.1354 (4)	0.0551 (4)	0.38612 (11)	0.0501 (11)	
C111	0.0696 (4)	0.1168 (4)	0.35991 (10)	0.0467 (11)	
C112	0.0135 (5)	0.0459 (4)	0.33201 (11)	0.0608 (13)	
H112	0.0133	−0.0382	0.3309	0.073*	
C113	−0.0416 (5)	0.0984 (5)	0.30600 (12)	0.0707 (14)	
H113	−0.0778	0.0504	0.2873	0.085*	
C114	−0.0425 (5)	0.2222 (5)	0.30788 (12)	0.0665 (14)	
C115	0.0082 (5)	0.2946 (4)	0.33513 (13)	0.0690 (14)	
H115	0.0052	0.3786	0.3361	0.083*	
C116	0.0638 (4)	0.2421 (4)	0.36114 (11)	0.0614 (13)	
H116	0.0979	0.2913	0.3798	0.074*	

### Atomic displacement parameters (Å^2^)

**Table:**

	*U*^11^	*U*^22^	*U*^33^	*U*^12^	*U*^13^	*U*^23^
S1	0.0443 (7)	0.0687 (8)	0.0495 (7)	0.0062 (6)	0.0026 (5)	−0.0020 (6)
S2	0.0463 (7)	0.0730 (8)	0.0533 (7)	−0.0078 (6)	0.0113 (6)	−0.0007 (6)
S3	0.0434 (7)	0.0755 (9)	0.0520 (7)	−0.0070 (6)	0.0014 (5)	0.0147 (6)
S4	0.0492 (7)	0.0738 (9)	0.0550 (7)	0.0076 (6)	0.0096 (6)	0.0142 (6)
Cl1	0.1194 (14)	0.1273 (14)	0.0909 (12)	−0.0069 (11)	0.0413 (10)	−0.0355 (10)
Cl2	0.0963 (11)	0.0992 (11)	0.0664 (9)	−0.0146 (9)	−0.0021 (8)	−0.0074 (8)
Cl3	0.1045 (12)	0.1482 (15)	0.0782 (11)	0.0078 (11)	0.0266 (9)	0.0498 (10)
Cl4	0.1242 (14)	0.1077 (12)	0.0787 (11)	0.0035 (10)	−0.0172 (9)	0.0288 (9)
O1	0.073 (2)	0.050 (2)	0.077 (2)	0.0054 (17)	0.0175 (18)	0.0164 (17)
O2	0.069 (2)	0.049 (2)	0.080 (2)	0.0000 (16)	0.0094 (18)	0.0180 (17)
O3	0.074 (2)	0.045 (2)	0.073 (2)	−0.0001 (17)	0.0129 (18)	0.0022 (16)
O4	0.072 (2)	0.048 (2)	0.080 (2)	−0.0001 (17)	0.0017 (18)	0.0042 (17)
N1	0.041 (2)	0.048 (2)	0.045 (2)	−0.0026 (16)	0.0011 (17)	0.0085 (17)
N2	0.045 (2)	0.054 (2)	0.053 (2)	0.0075 (18)	0.0102 (18)	0.0140 (19)
N3	0.038 (2)	0.046 (2)	0.047 (2)	0.0000 (16)	0.0005 (17)	0.0034 (17)
N4	0.046 (2)	0.052 (2)	0.048 (2)	−0.0023 (17)	0.0098 (18)	0.0003 (18)
C1	0.036 (2)	0.049 (3)	0.049 (3)	−0.002 (2)	0.004 (2)	0.014 (2)
C2	0.051 (3)	0.050 (3)	0.053 (3)	−0.006 (2)	0.002 (2)	0.012 (2)
C3	0.044 (3)	0.068 (3)	0.066 (3)	−0.002 (2)	−0.006 (2)	0.017 (3)
C4	0.045 (3)	0.066 (4)	0.107 (5)	0.009 (3)	0.010 (3)	0.030 (3)
C5	0.060 (4)	0.067 (4)	0.104 (5)	0.019 (3)	0.023 (3)	0.021 (3)
C6	0.068 (4)	0.073 (4)	0.104 (5)	0.015 (3)	0.019 (4)	0.002 (3)
C7	0.048 (3)	0.076 (4)	0.070 (3)	0.009 (3)	0.008 (3)	0.003 (3)
C8	0.042 (3)	0.050 (3)	0.061 (3)	0.000 (2)	0.012 (2)	0.011 (2)
C9	0.045 (3)	0.052 (3)	0.067 (3)	0.001 (2)	0.009 (2)	0.018 (2)
C10	0.056 (3)	0.053 (3)	0.050 (3)	0.000 (2)	−0.006 (2)	0.014 (2)
C11	0.067 (3)	0.073 (4)	0.066 (3)	−0.005 (3)	−0.019 (3)	0.000 (3)
C12	0.100 (5)	0.070 (4)	0.071 (4)	0.005 (3)	−0.030 (4)	−0.005 (3)
C13	0.112 (5)	0.066 (4)	0.051 (3)	0.025 (4)	−0.012 (4)	−0.001 (3)
C14	0.099 (5)	0.078 (4)	0.052 (3)	0.016 (3)	0.006 (3)	0.009 (3)
C15	0.071 (4)	0.069 (3)	0.053 (3)	−0.004 (3)	0.004 (3)	0.011 (3)
C16	0.046 (3)	0.048 (3)	0.047 (3)	0.000 (2)	0.006 (2)	0.007 (2)
C17	0.045 (3)	0.060 (3)	0.044 (3)	0.009 (2)	0.002 (2)	0.008 (2)
C18	0.061 (3)	0.069 (3)	0.056 (3)	0.013 (3)	0.012 (3)	0.012 (3)
C19	0.100 (5)	0.093 (4)	0.052 (3)	0.034 (4)	0.016 (3)	0.026 (3)
C20	0.091 (5)	0.117 (6)	0.062 (4)	0.026 (4)	−0.016 (3)	0.004 (4)
C21	0.077 (4)	0.104 (5)	0.089 (5)	−0.001 (4)	−0.028 (4)	0.008 (4)
C22	0.063 (4)	0.083 (4)	0.078 (4)	−0.006 (3)	−0.012 (3)	0.020 (3)
C23	0.037 (2)	0.054 (3)	0.056 (3)	0.002 (2)	0.002 (2)	0.014 (2)
C24	0.041 (3)	0.057 (3)	0.042 (3)	0.004 (2)	0.007 (2)	0.017 (2)
C25	0.071 (3)	0.063 (3)	0.052 (3)	0.013 (3)	0.017 (3)	0.016 (2)
C26	0.083 (4)	0.062 (3)	0.081 (4)	0.021 (3)	0.026 (3)	0.008 (3)
C27	0.059 (3)	0.081 (4)	0.070 (4)	−0.003 (3)	0.013 (3)	−0.009 (3)
C28	0.081 (4)	0.088 (4)	0.057 (3)	−0.010 (3)	0.021 (3)	0.007 (3)
C29	0.063 (3)	0.068 (3)	0.062 (3)	−0.005 (3)	0.010 (3)	0.016 (3)
C30	0.044 (3)	0.049 (3)	0.050 (3)	0.005 (2)	0.005 (2)	0.012 (2)
C31	0.049 (3)	0.060 (3)	0.056 (3)	0.015 (2)	0.016 (2)	0.019 (2)
C32	0.050 (3)	0.070 (3)	0.078 (4)	0.007 (3)	0.023 (3)	0.021 (3)
C33	0.050 (3)	0.068 (3)	0.088 (4)	−0.004 (3)	0.015 (3)	0.023 (3)
C34	0.051 (3)	0.069 (4)	0.109 (5)	−0.008 (3)	0.005 (3)	0.021 (3)
C35	0.069 (4)	0.073 (4)	0.089 (4)	−0.016 (3)	0.005 (3)	−0.003 (3)
C36	0.049 (3)	0.074 (4)	0.084 (4)	−0.010 (3)	0.009 (3)	0.002 (3)
C37	0.042 (3)	0.052 (3)	0.060 (3)	0.001 (2)	0.004 (2)	0.018 (2)
C38	0.041 (3)	0.057 (3)	0.073 (3)	0.002 (2)	0.011 (2)	0.021 (3)
C39	0.055 (3)	0.056 (3)	0.051 (3)	0.011 (2)	0.017 (2)	0.013 (2)
C40	0.064 (3)	0.095 (4)	0.072 (4)	0.004 (3)	0.026 (3)	−0.003 (3)
C41	0.083 (4)	0.094 (4)	0.075 (4)	0.014 (3)	0.030 (3)	−0.007 (3)
C42	0.092 (4)	0.075 (4)	0.055 (3)	−0.002 (3)	0.017 (3)	0.002 (3)
C43	0.066 (3)	0.087 (4)	0.060 (3)	0.009 (3)	0.004 (3)	0.008 (3)
C44	0.061 (3)	0.078 (4)	0.052 (3)	0.016 (3)	0.011 (3)	0.005 (3)
C45	0.043 (3)	0.051 (3)	0.051 (3)	0.001 (2)	0.010 (2)	0.009 (2)
C46	0.044 (3)	0.055 (3)	0.044 (3)	−0.008 (2)	0.006 (2)	0.003 (2)
C47	0.062 (3)	0.066 (3)	0.060 (3)	−0.008 (3)	0.001 (3)	0.016 (3)
C48	0.093 (5)	0.102 (5)	0.054 (3)	−0.036 (4)	−0.005 (3)	0.027 (3)
C49	0.100 (5)	0.124 (6)	0.050 (4)	−0.037 (4)	0.026 (3)	0.000 (4)
C50	0.092 (5)	0.108 (5)	0.087 (5)	0.005 (4)	0.050 (4)	0.013 (4)
C51	0.076 (4)	0.083 (4)	0.083 (4)	0.012 (3)	0.034 (3)	0.020 (3)
C52	0.042 (3)	0.050 (3)	0.051 (3)	0.000 (2)	0.013 (2)	0.015 (2)
C53	0.040 (2)	0.052 (3)	0.043 (3)	0.004 (2)	0.008 (2)	0.012 (2)
C54	0.063 (3)	0.056 (3)	0.055 (3)	−0.002 (2)	−0.001 (2)	0.021 (2)
C55	0.073 (3)	0.059 (3)	0.061 (3)	−0.012 (3)	−0.001 (3)	0.019 (3)
C56	0.050 (3)	0.076 (4)	0.056 (3)	−0.003 (3)	0.012 (2)	0.009 (3)
C57	0.073 (3)	0.074 (4)	0.046 (3)	0.016 (3)	0.008 (3)	0.019 (3)
C58	0.059 (3)	0.055 (3)	0.054 (3)	0.010 (2)	0.009 (2)	0.015 (2)
C59	0.040 (3)	0.043 (3)	0.055 (3)	0.005 (2)	0.003 (2)	0.000 (2)
C60	0.041 (3)	0.048 (3)	0.060 (3)	0.000 (2)	−0.001 (2)	−0.003 (2)
C61	0.042 (3)	0.064 (3)	0.069 (3)	0.000 (2)	−0.007 (2)	0.002 (3)
C62	0.041 (3)	0.065 (3)	0.084 (4)	−0.009 (2)	0.006 (3)	−0.007 (3)
C63	0.050 (3)	0.065 (4)	0.112 (5)	−0.008 (3)	0.023 (3)	0.012 (3)
C64	0.065 (4)	0.083 (4)	0.089 (4)	−0.010 (3)	0.020 (3)	0.025 (3)
C65	0.053 (3)	0.078 (4)	0.078 (4)	−0.010 (3)	0.007 (3)	0.019 (3)
C66	0.043 (3)	0.046 (3)	0.063 (3)	−0.001 (2)	0.008 (2)	0.000 (2)
C67	0.038 (3)	0.048 (3)	0.071 (3)	0.000 (2)	0.006 (2)	−0.003 (2)
C68	0.051 (3)	0.046 (3)	0.049 (3)	0.003 (2)	−0.008 (2)	0.002 (2)
C69	0.058 (3)	0.091 (4)	0.086 (4)	−0.003 (3)	−0.013 (3)	0.029 (3)
C70	0.067 (4)	0.099 (5)	0.089 (4)	0.002 (3)	−0.023 (3)	0.037 (4)
C71	0.086 (4)	0.058 (3)	0.059 (3)	−0.001 (3)	−0.005 (3)	0.007 (2)
C72	0.066 (3)	0.075 (4)	0.062 (3)	0.010 (3)	0.007 (3)	0.007 (3)
C73	0.049 (3)	0.067 (3)	0.057 (3)	0.009 (2)	−0.004 (2)	0.009 (2)
C74	0.043 (3)	0.049 (3)	0.048 (3)	0.000 (2)	0.004 (2)	0.007 (2)
C75	0.047 (3)	0.057 (3)	0.042 (3)	−0.011 (2)	0.009 (2)	0.008 (2)
C76	0.064 (4)	0.082 (4)	0.078 (4)	0.001 (3)	−0.019 (3)	0.004 (3)
C77	0.080 (4)	0.111 (5)	0.090 (5)	−0.003 (4)	−0.027 (4)	0.015 (4)
C78	0.090 (5)	0.125 (6)	0.065 (4)	−0.038 (4)	−0.022 (4)	0.011 (4)
C79	0.111 (5)	0.092 (4)	0.052 (4)	−0.044 (4)	0.013 (3)	−0.013 (3)
C80	0.067 (3)	0.067 (3)	0.052 (3)	−0.009 (3)	0.011 (3)	0.001 (3)
C81	0.036 (2)	0.050 (3)	0.052 (3)	−0.005 (2)	−0.003 (2)	−0.002 (2)
C82	0.036 (2)	0.047 (3)	0.049 (3)	−0.001 (2)	0.006 (2)	−0.002 (2)
C83	0.061 (3)	0.058 (3)	0.058 (3)	−0.003 (2)	0.009 (2)	0.002 (2)
C84	0.078 (4)	0.061 (3)	0.067 (4)	−0.016 (3)	0.012 (3)	0.013 (3)
C85	0.051 (3)	0.088 (4)	0.053 (3)	−0.002 (3)	0.009 (2)	0.012 (3)
C86	0.070 (4)	0.092 (4)	0.044 (3)	0.014 (3)	0.009 (3)	0.001 (3)
C87	0.059 (3)	0.054 (3)	0.057 (3)	0.010 (2)	0.002 (2)	−0.003 (2)
C88	0.041 (3)	0.049 (3)	0.046 (3)	−0.006 (2)	0.007 (2)	0.001 (2)
C89	0.051 (3)	0.051 (3)	0.056 (3)	−0.006 (2)	0.015 (2)	−0.001 (2)
C90	0.060 (3)	0.064 (3)	0.067 (3)	0.000 (3)	0.023 (3)	0.001 (3)
C91	0.043 (3)	0.070 (4)	0.096 (4)	0.005 (3)	0.012 (3)	0.001 (3)
C92	0.058 (4)	0.063 (4)	0.118 (5)	0.019 (3)	0.008 (4)	0.013 (3)
C93	0.064 (4)	0.077 (4)	0.102 (5)	0.017 (3)	0.002 (3)	0.026 (3)
C94	0.059 (3)	0.071 (3)	0.073 (4)	0.011 (3)	0.007 (3)	0.013 (3)
C95	0.042 (3)	0.048 (3)	0.062 (3)	−0.002 (2)	0.003 (2)	0.002 (2)
C96	0.046 (3)	0.048 (3)	0.070 (3)	−0.004 (2)	0.011 (2)	−0.002 (2)
C97	0.065 (3)	0.050 (3)	0.051 (3)	0.004 (2)	0.017 (3)	−0.003 (2)
C98	0.074 (4)	0.080 (4)	0.065 (3)	0.004 (3)	0.026 (3)	0.014 (3)
C99	0.119 (6)	0.081 (4)	0.067 (4)	0.008 (4)	0.044 (4)	0.016 (3)
C100	0.117 (5)	0.084 (4)	0.056 (4)	0.028 (4)	0.019 (4)	0.011 (3)
C101	0.092 (5)	0.090 (4)	0.068 (4)	0.013 (4)	0.004 (3)	0.003 (3)
C102	0.071 (4)	0.076 (4)	0.060 (3)	0.000 (3)	0.006 (3)	0.013 (3)
C103	0.043 (3)	0.049 (3)	0.049 (3)	−0.002 (2)	0.005 (2)	0.007 (2)
C104	0.049 (3)	0.055 (3)	0.048 (3)	0.009 (2)	0.002 (2)	0.005 (2)
C105	0.065 (3)	0.065 (3)	0.052 (3)	0.010 (3)	−0.001 (3)	0.002 (2)
C106	0.100 (5)	0.088 (4)	0.055 (4)	0.026 (4)	−0.005 (3)	−0.008 (3)
C107	0.106 (5)	0.125 (6)	0.065 (4)	0.045 (5)	0.031 (4)	0.016 (4)
C108	0.106 (5)	0.105 (5)	0.094 (5)	0.011 (4)	0.054 (4)	0.016 (4)
C109	0.080 (4)	0.075 (4)	0.075 (4)	−0.003 (3)	0.027 (3)	0.005 (3)
C110	0.042 (3)	0.049 (3)	0.058 (3)	0.002 (2)	0.012 (2)	−0.004 (2)
C111	0.043 (3)	0.047 (3)	0.047 (3)	0.000 (2)	0.005 (2)	−0.004 (2)
C112	0.071 (3)	0.053 (3)	0.055 (3)	−0.013 (2)	0.008 (3)	−0.004 (2)
C113	0.077 (4)	0.071 (4)	0.059 (3)	−0.012 (3)	0.002 (3)	−0.006 (3)
C114	0.058 (3)	0.070 (4)	0.070 (4)	0.000 (3)	0.000 (3)	0.008 (3)
C115	0.074 (4)	0.049 (3)	0.079 (4)	0.012 (3)	−0.006 (3)	0.003 (3)
C116	0.066 (3)	0.053 (3)	0.058 (3)	0.002 (2)	−0.008 (3)	−0.007 (2)

### Geometric parameters (Å, °)

**Table:**

S1—C1	1.770 (4)	C51—H51	0.9300
S1—C16	1.807 (4)	C52—C53	1.487 (6)
S2—C30	1.763 (4)	C53—C58	1.380 (6)
S2—C45	1.809 (4)	C53—C54	1.384 (5)
S3—C59	1.769 (4)	C54—C55	1.382 (6)
S3—C74	1.805 (4)	C54—H54	0.9300
S4—C88	1.769 (4)	C55—C56	1.367 (6)
S4—C103	1.806 (4)	C55—H55	0.9300
Cl1—C27	1.740 (5)	C56—C57	1.379 (6)
Cl2—C56	1.728 (5)	C57—C58	1.390 (6)
Cl3—C85	1.735 (5)	C57—H57	0.9300
Cl4—C114	1.735 (5)	C58—H58	0.9300
O1—C23	1.217 (5)	C59—C66	1.428 (6)
O2—C52	1.217 (5)	C60—C61	1.359 (6)
O3—C81	1.214 (5)	C60—C68	1.488 (6)
O4—C110	1.218 (5)	C61—C67	1.413 (6)
N1—C1	1.310 (5)	C61—H61	0.9300
N1—C2	1.383 (5)	C62—C63	1.357 (7)
N2—C30	1.311 (5)	C62—C67	1.407 (6)
N2—C31	1.389 (5)	C62—H62	0.9300
N3—C59	1.309 (5)	C63—C64	1.386 (7)
N3—C60	1.380 (5)	C63—H63	0.9300
N4—C88	1.322 (5)	C64—C65	1.360 (6)
N4—C89	1.380 (5)	C64—H64	0.9300
C1—C8	1.430 (5)	C65—C66	1.407 (6)
C2—C3	1.364 (6)	C65—H65	0.9300
C2—C10	1.475 (6)	C66—C67	1.416 (6)
C3—C9	1.419 (6)	C68—C73	1.380 (6)
C3—H3	0.9300	C68—C69	1.385 (6)
C4—C5	1.351 (7)	C69—C70	1.382 (7)
C4—C9	1.418 (6)	C69—H69	0.9300
C4—H4	0.9300	C70—C71	1.361 (7)
C5—C6	1.412 (7)	C70—H70	0.9300
C5—H5	0.9300	C71—C72	1.371 (6)
C6—C7	1.355 (6)	C71—H71	0.9300
C6—H6	0.9300	C72—C73	1.370 (6)
C7—C8	1.408 (6)	C72—H72	0.9300
C7—H7	0.9300	C73—H73	0.9300
C8—C9	1.406 (6)	C74—C81	1.517 (6)
C10—C11	1.382 (6)	C74—C75	1.515 (6)
C10—C15	1.388 (6)	C74—H74	0.9800
C11—C12	1.387 (7)	C75—C76	1.372 (6)
C11—H11	0.9300	C75—C80	1.384 (6)
C12—C13	1.355 (7)	C76—C77	1.387 (7)
C12—H12	0.9300	C76—H76	0.9300
C13—C14	1.355 (7)	C77—C78	1.337 (8)
C13—H13	0.9300	C77—H77	0.9300
C14—C15	1.386 (7)	C78—C79	1.366 (8)
C14—H14	0.9300	C78—H78	0.9300
C15—H15	0.9300	C79—C80	1.394 (7)
C16—C17	1.522 (5)	C79—H79	0.9300
C16—C23	1.534 (6)	C80—H80	0.9300
C16—H16	0.9800	C81—C82	1.496 (6)
C17—C22	1.377 (6)	C82—C87	1.375 (6)
C17—C18	1.382 (6)	C82—C83	1.386 (6)
C18—C19	1.389 (7)	C83—C84	1.371 (6)
C18—H18	0.9300	C83—H83	0.9300
C19—C20	1.365 (8)	C84—C85	1.367 (7)
C19—H19	0.9300	C84—H84	0.9300
C20—C21	1.358 (8)	C85—C86	1.362 (6)
C20—H20	0.9300	C86—C87	1.394 (6)
C21—C22	1.378 (7)	C86—H86	0.9300
C21—H21	0.9300	C87—H87	0.9300
C22—H22	0.9300	C88—C95	1.429 (6)
C23—C24	1.482 (6)	C89—C90	1.373 (6)
C24—C25	1.378 (6)	C89—C97	1.470 (6)
C24—C29	1.386 (6)	C90—C96	1.402 (6)
C25—C26	1.390 (6)	C90—H90	0.9300
C25—H25	0.9300	C91—C92	1.346 (7)
C26—C27	1.359 (7)	C91—C96	1.415 (6)
C26—H26	0.9300	C91—H91	0.9300
C27—C28	1.374 (7)	C92—C93	1.400 (7)
C28—C29	1.379 (6)	C92—H92	0.9300
C28—H28	0.9300	C93—C94	1.373 (6)
C29—H29	0.9300	C93—H93	0.9300
C30—C37	1.430 (6)	C94—C95	1.397 (6)
C31—C32	1.369 (6)	C94—H94	0.9300
C31—C39	1.473 (6)	C95—C96	1.410 (6)
C32—C38	1.405 (6)	C97—C102	1.383 (6)
C32—H32	0.9300	C97—C98	1.395 (6)
C33—C34	1.350 (7)	C98—C99	1.388 (7)
C33—C38	1.406 (6)	C98—H98	0.9300
C33—H33	0.9300	C99—C100	1.389 (8)
C34—C35	1.401 (7)	C99—H99	0.9300
C34—H34	0.9300	C100—C101	1.368 (8)
C35—C36	1.369 (6)	C100—H100	0.9300
C35—H35	0.9300	C101—C102	1.363 (7)
C36—C37	1.409 (6)	C101—H101	0.9300
C36—H36	0.9300	C102—H102	0.9300
C37—C38	1.412 (6)	C103—C104	1.516 (6)
C39—C44	1.374 (6)	C103—C110	1.537 (6)
C39—C40	1.391 (6)	C103—H103	0.9800
C40—C41	1.367 (7)	C104—C109	1.379 (6)
C40—H40	0.9300	C104—C105	1.385 (6)
C41—C42	1.379 (7)	C105—C106	1.395 (7)
C41—H41	0.9300	C105—H105	0.9300
C42—C43	1.375 (6)	C106—C107	1.358 (8)
C42—H42	0.9300	C106—H106	0.9300
C43—C44	1.377 (6)	C107—C108	1.360 (8)
C43—H43	0.9300	C107—H107	0.9300
C44—H44	0.9300	C108—C109	1.385 (7)
C45—C46	1.518 (5)	C108—H108	0.9300
C45—C52	1.519 (6)	C109—H109	0.9300
C45—H45	0.9800	C110—C111	1.475 (6)
C46—C51	1.382 (6)	C111—C116	1.388 (6)
C46—C47	1.385 (6)	C111—C112	1.392 (6)
C47—C48	1.391 (7)	C112—C113	1.375 (6)
C47—H47	0.9300	C112—H112	0.9300
C48—C49	1.372 (8)	C113—C114	1.368 (6)
C48—H48	0.9300	C113—H113	0.9300
C49—C50	1.359 (8)	C114—C115	1.362 (6)
C49—H49	0.9300	C115—C116	1.376 (6)
C50—C51	1.373 (7)	C115—H115	0.9300
C50—H50	0.9300	C116—H116	0.9300
			
C1—S1—C16	102.4 (2)	C58—C57—H57	120.6
C30—S2—C45	102.6 (2)	C53—C58—C57	121.4 (4)
C59—S3—C74	103.3 (2)	C53—C58—H58	119.3
C88—S4—C103	102.6 (2)	C57—C58—H58	119.3
C1—N1—C2	117.8 (4)	N3—C59—C66	125.1 (4)
C30—N2—C31	118.9 (4)	N3—C59—S3	119.2 (3)
C59—N3—C60	118.3 (4)	C66—C59—S3	115.7 (3)
C88—N4—C89	117.8 (4)	C61—C60—N3	120.8 (4)
N1—C1—C8	125.0 (4)	C61—C60—C68	123.6 (4)
N1—C1—S1	119.0 (3)	N3—C60—C68	115.6 (4)
C8—C1—S1	116.0 (3)	C60—C61—C67	122.1 (4)
C3—C2—N1	121.7 (4)	C60—C61—H61	118.9
C3—C2—C10	122.8 (4)	C67—C61—H61	118.9
N1—C2—C10	115.6 (4)	C63—C62—C67	120.6 (5)
C2—C3—C9	120.9 (4)	C63—C62—H62	119.7
C2—C3—H3	119.5	C67—C62—H62	119.7
C9—C3—H3	119.5	C62—C63—C64	120.8 (5)
C5—C4—C9	119.4 (5)	C62—C63—H63	119.6
C5—C4—H4	120.3	C64—C63—H63	119.6
C9—C4—H4	120.3	C65—C64—C63	120.6 (5)
C4—C5—C6	121.8 (5)	C65—C64—H64	119.7
C4—C5—H5	119.1	C63—C64—H64	119.7
C6—C5—H5	119.1	C64—C65—C66	120.1 (5)
C7—C6—C5	119.2 (5)	C64—C65—H65	119.9
C7—C6—H6	120.4	C66—C65—H65	119.9
C5—C6—H6	120.4	C65—C66—C67	119.3 (4)
C6—C7—C8	121.1 (5)	C65—C66—C59	124.3 (4)
C6—C7—H7	119.4	C67—C66—C59	116.4 (4)
C8—C7—H7	119.4	C62—C67—C61	124.4 (5)
C7—C8—C9	119.1 (4)	C62—C67—C66	118.4 (5)
C7—C8—C1	124.3 (4)	C61—C67—C66	117.2 (4)
C9—C8—C1	116.6 (4)	C73—C68—C69	117.4 (5)
C8—C9—C3	117.8 (4)	C73—C68—C60	121.1 (4)
C8—C9—C4	119.4 (5)	C69—C68—C60	121.4 (4)
C3—C9—C4	122.8 (5)	C70—C69—C68	121.1 (5)
C11—C10—C15	117.8 (5)	C70—C69—H69	119.5
C11—C10—C2	120.8 (5)	C68—C69—H69	119.5
C15—C10—C2	121.4 (4)	C71—C70—C69	120.7 (5)
C10—C11—C12	120.0 (5)	C71—C70—H70	119.7
C10—C11—H11	120.0	C69—C70—H70	119.7
C12—C11—H11	120.0	C70—C71—C72	118.6 (5)
C13—C12—C11	121.1 (6)	C70—C71—H71	120.7
C13—C12—H12	119.5	C72—C71—H71	120.7
C11—C12—H12	119.5	C73—C72—C71	121.4 (5)
C14—C13—C12	120.1 (5)	C73—C72—H72	119.3
C14—C13—H13	120.0	C71—C72—H72	119.3
C12—C13—H13	120.0	C72—C73—C68	120.8 (4)
C13—C14—C15	119.8 (6)	C72—C73—H73	119.6
C13—C14—H14	120.1	C68—C73—H73	119.6
C15—C14—H14	120.1	C81—C74—C75	112.1 (3)
C14—C15—C10	121.1 (5)	C81—C74—S3	111.7 (3)
C14—C15—H15	119.4	C75—C74—S3	105.5 (3)
C10—C15—H15	119.4	C81—C74—H74	109.2
C17—C16—C23	112.3 (3)	C75—C74—H74	109.2
C17—C16—S1	105.0 (3)	S3—C74—H74	109.2
C23—C16—S1	111.6 (3)	C76—C75—C80	117.9 (4)
C17—C16—H16	109.3	C76—C75—C74	122.4 (4)
C23—C16—H16	109.3	C80—C75—C74	119.7 (4)
S1—C16—H16	109.3	C75—C76—C77	120.7 (5)
C22—C17—C18	118.0 (4)	C75—C76—H76	119.7
C22—C17—C16	122.3 (4)	C77—C76—H76	119.7
C18—C17—C16	119.6 (4)	C78—C77—C76	121.6 (6)
C17—C18—C19	120.0 (5)	C78—C77—H77	119.2
C17—C18—H18	120.0	C76—C77—H77	119.2
C19—C18—H18	120.0	C77—C78—C79	118.9 (6)
C20—C19—C18	120.7 (5)	C77—C78—H78	120.6
C20—C19—H19	119.7	C79—C78—H78	120.6
C18—C19—H19	119.7	C78—C79—C80	120.9 (6)
C21—C20—C19	119.7 (6)	C78—C79—H79	119.5
C21—C20—H20	120.1	C80—C79—H79	119.5
C19—C20—H20	120.1	C75—C80—C79	120.0 (5)
C20—C21—C22	119.9 (6)	C75—C80—H80	120.0
C20—C21—H21	120.1	C79—C80—H80	120.0
C22—C21—H21	120.1	O3—C81—C82	120.3 (4)
C21—C22—C17	121.6 (5)	O3—C81—C74	120.9 (4)
C21—C22—H22	119.2	C82—C81—C74	118.8 (4)
C17—C22—H22	119.2	C87—C82—C83	118.8 (4)
O1—C23—C24	121.2 (4)	C87—C82—C81	118.4 (4)
O1—C23—C16	120.3 (4)	C83—C82—C81	122.8 (4)
C24—C23—C16	118.5 (4)	C84—C83—C82	120.7 (4)
C25—C24—C29	118.9 (4)	C84—C83—H83	119.6
C25—C24—C23	123.2 (4)	C82—C83—H83	119.6
C29—C24—C23	117.8 (4)	C83—C84—C85	119.5 (5)
C24—C25—C26	120.6 (4)	C83—C84—H84	120.2
C24—C25—H25	119.7	C85—C84—H84	120.2
C26—C25—H25	119.7	C86—C85—C84	121.4 (5)
C27—C26—C25	118.8 (5)	C86—C85—Cl3	119.1 (4)
C27—C26—H26	120.6	C84—C85—Cl3	119.5 (4)
C25—C26—H26	120.6	C85—C86—C87	118.9 (4)
C26—C27—C28	122.3 (5)	C85—C86—H86	120.6
C26—C27—Cl1	118.9 (5)	C87—C86—H86	120.6
C28—C27—Cl1	118.7 (5)	C82—C87—C86	120.6 (5)
C27—C28—C29	118.3 (5)	C82—C87—H87	119.7
C27—C28—H28	120.9	C86—C87—H87	119.7
C29—C28—H28	120.9	N4—C88—C95	124.8 (4)
C28—C29—C24	121.0 (5)	N4—C88—S4	118.6 (3)
C28—C29—H29	119.5	C95—C88—S4	116.5 (3)
C24—C29—H29	119.5	C90—C89—N4	121.5 (4)
N2—C30—C37	124.3 (4)	C90—C89—C97	123.3 (4)
N2—C30—S2	119.0 (3)	N4—C89—C97	115.1 (4)
C37—C30—S2	116.7 (3)	C89—C90—C96	121.0 (4)
C32—C31—N2	120.2 (4)	C89—C90—H90	119.5
C32—C31—C39	123.8 (4)	C96—C90—H90	119.5
N2—C31—C39	116.0 (4)	C92—C91—C96	121.0 (5)
C31—C32—C38	122.0 (4)	C92—C91—H91	119.5
C31—C32—H32	119.0	C96—C91—H91	119.5
C38—C32—H32	119.0	C91—C92—C93	120.9 (5)
C34—C33—C38	120.4 (5)	C91—C92—H92	119.5
C34—C33—H33	119.8	C93—C92—H92	119.5
C38—C33—H33	119.8	C94—C93—C92	119.8 (5)
C33—C34—C35	121.5 (5)	C94—C93—H93	120.1
C33—C34—H34	119.2	C92—C93—H93	120.1
C35—C34—H34	119.2	C93—C94—C95	120.4 (5)
C36—C35—C34	119.7 (5)	C93—C94—H94	119.8
C36—C35—H35	120.1	C95—C94—H94	119.8
C34—C35—H35	120.1	C94—C95—C96	119.9 (4)
C35—C36—C37	119.9 (5)	C94—C95—C88	123.8 (4)
C35—C36—H36	120.0	C96—C95—C88	116.3 (4)
C37—C36—H36	120.0	C90—C96—C95	118.5 (4)
C36—C37—C38	119.8 (4)	C90—C96—C91	123.3 (5)
C36—C37—C30	123.3 (4)	C95—C96—C91	118.2 (5)
C38—C37—C30	116.9 (4)	C102—C97—C98	117.4 (5)
C33—C38—C32	123.7 (5)	C102—C97—C89	121.8 (4)
C33—C38—C37	118.6 (5)	C98—C97—C89	120.8 (5)
C32—C38—C37	117.7 (4)	C99—C98—C97	120.6 (5)
C44—C39—C40	117.2 (5)	C99—C98—H98	119.7
C44—C39—C31	120.9 (4)	C97—C98—H98	119.7
C40—C39—C31	121.9 (4)	C98—C99—C100	120.4 (6)
C41—C40—C39	120.8 (5)	C98—C99—H99	119.8
C41—C40—H40	119.6	C100—C99—H99	119.8
C39—C40—H40	119.6	C101—C100—C99	118.5 (6)
C40—C41—C42	121.7 (5)	C101—C100—H100	120.7
C40—C41—H41	119.1	C99—C100—H100	120.7
C42—C41—H41	119.1	C102—C101—C100	121.2 (6)
C43—C42—C41	117.7 (5)	C102—C101—H101	119.4
C43—C42—H42	121.1	C100—C101—H101	119.4
C41—C42—H42	121.1	C101—C102—C97	121.8 (5)
C42—C43—C44	120.7 (5)	C101—C102—H102	119.1
C42—C43—H43	119.7	C97—C102—H102	119.1
C44—C43—H43	119.7	C104—C103—C110	111.8 (3)
C39—C44—C43	121.8 (5)	C104—C103—S4	105.8 (3)
C39—C44—H44	119.1	C110—C103—S4	111.6 (3)
C43—C44—H44	119.1	C104—C103—H103	109.2
C46—C45—C52	111.6 (3)	C110—C103—H103	109.2
C46—C45—S2	105.7 (3)	S4—C103—H103	109.2
C52—C45—S2	111.9 (3)	C109—C104—C105	118.0 (5)
C46—C45—H45	109.2	C109—C104—C103	122.2 (4)
C52—C45—H45	109.2	C105—C104—C103	119.7 (4)
S2—C45—H45	109.2	C104—C105—C106	120.2 (5)
C51—C46—C47	117.6 (4)	C104—C105—H105	119.9
C51—C46—C45	122.2 (4)	C106—C105—H105	119.9
C47—C46—C45	120.1 (4)	C107—C106—C105	120.4 (6)
C46—C47—C48	120.7 (5)	C107—C106—H106	119.8
C46—C47—H47	119.6	C105—C106—H106	119.8
C48—C47—H47	119.6	C108—C107—C106	120.3 (6)
C49—C48—C47	120.0 (5)	C108—C107—H107	119.9
C49—C48—H48	120.0	C106—C107—H107	119.9
C47—C48—H48	120.0	C107—C108—C109	119.8 (6)
C50—C49—C48	119.6 (5)	C107—C108—H108	120.1
C50—C49—H49	120.2	C109—C108—H108	120.1
C48—C49—H49	120.2	C104—C109—C108	121.4 (5)
C49—C50—C51	120.7 (6)	C104—C109—H109	119.3
C49—C50—H50	119.7	C108—C109—H109	119.3
C51—C50—H50	119.7	O4—C110—C111	121.3 (4)
C50—C51—C46	121.3 (5)	O4—C110—C103	120.1 (4)
C50—C51—H51	119.3	C111—C110—C103	118.7 (4)
C46—C51—H51	119.3	C116—C111—C112	118.0 (4)
O2—C52—C53	120.4 (4)	C116—C111—C110	123.6 (4)
O2—C52—C45	121.0 (4)	C112—C111—C110	118.4 (4)
C53—C52—C45	118.6 (4)	C113—C112—C111	120.9 (5)
C58—C53—C54	118.7 (4)	C113—C112—H112	119.5
C58—C53—C52	118.7 (4)	C111—C112—H112	119.5
C54—C53—C52	122.5 (4)	C114—C113—C112	119.2 (5)
C55—C54—C53	120.1 (4)	C114—C113—H113	120.4
C55—C54—H54	119.9	C112—C113—H113	120.4
C53—C54—H54	119.9	C115—C114—C113	121.5 (5)
C56—C55—C54	120.5 (5)	C115—C114—Cl4	119.0 (4)
C56—C55—H55	119.7	C113—C114—Cl4	119.5 (4)
C54—C55—H55	119.7	C114—C115—C116	119.3 (5)
C55—C56—C57	120.5 (5)	C114—C115—H115	120.3
C55—C56—Cl2	120.1 (4)	C116—C115—H115	120.3
C57—C56—Cl2	119.4 (4)	C115—C116—C111	121.0 (4)
C56—C57—C58	118.7 (4)	C115—C116—H116	119.5
C56—C57—H57	120.6	C111—C116—H116	119.5
			
C2—N1—C1—C8	3.4 (6)	C60—N3—C59—C66	2.4 (6)
C2—N1—C1—S1	−176.2 (3)	C60—N3—C59—S3	−176.2 (3)
C16—S1—C1—N1	−1.2 (4)	C74—S3—C59—N3	−2.1 (4)
C16—S1—C1—C8	179.2 (3)	C74—S3—C59—C66	179.2 (3)
C1—N1—C2—C3	−2.4 (6)	C59—N3—C60—C61	−1.2 (6)
C1—N1—C2—C10	178.4 (4)	C59—N3—C60—C68	179.5 (4)
N1—C2—C3—C9	0.6 (7)	N3—C60—C61—C67	−0.3 (7)
C10—C2—C3—C9	179.7 (4)	C68—C60—C61—C67	178.8 (4)
C9—C4—C5—C6	0.7 (8)	C67—C62—C63—C64	0.0 (8)
C4—C5—C6—C7	−0.8 (8)	C62—C63—C64—C65	1.5 (8)
C5—C6—C7—C8	0.6 (8)	C63—C64—C65—C66	−1.4 (8)
C6—C7—C8—C9	−0.5 (7)	C64—C65—C66—C67	−0.2 (7)
C6—C7—C8—C1	−179.8 (4)	C64—C65—C66—C59	−179.2 (5)
N1—C1—C8—C7	177.0 (4)	N3—C59—C66—C65	177.2 (4)
S1—C1—C8—C7	−3.4 (6)	S3—C59—C66—C65	−4.1 (6)
N1—C1—C8—C9	−2.4 (6)	N3—C59—C66—C67	−1.8 (6)
S1—C1—C8—C9	177.2 (3)	S3—C59—C66—C67	176.8 (3)
C7—C8—C9—C3	−179.0 (4)	C63—C62—C67—C61	179.1 (5)
C1—C8—C9—C3	0.4 (6)	C63—C62—C67—C66	−1.6 (7)
C7—C8—C9—C4	0.4 (6)	C60—C61—C67—C62	−179.9 (4)
C1—C8—C9—C4	179.8 (4)	C60—C61—C67—C66	0.9 (7)
C2—C3—C9—C8	0.4 (6)	C65—C66—C67—C62	1.7 (6)
C2—C3—C9—C4	−179.0 (4)	C59—C66—C67—C62	−179.2 (4)
C5—C4—C9—C8	−0.6 (7)	C65—C66—C67—C61	−179.0 (4)
C5—C4—C9—C3	178.9 (5)	C59—C66—C67—C61	0.1 (6)
C3—C2—C10—C11	34.8 (7)	C61—C60—C68—C73	−158.9 (4)
N1—C2—C10—C11	−146.1 (4)	N3—C60—C68—C73	20.3 (6)
C3—C2—C10—C15	−146.7 (5)	C61—C60—C68—C69	22.7 (7)
N1—C2—C10—C15	32.4 (6)	N3—C60—C68—C69	−158.1 (4)
C15—C10—C11—C12	2.6 (7)	C73—C68—C69—C70	2.3 (8)
C2—C10—C11—C12	−178.9 (4)	C60—C68—C69—C70	−179.2 (5)
C10—C11—C12—C13	−1.0 (8)	C68—C69—C70—C71	−1.6 (9)
C11—C12—C13—C14	−0.6 (8)	C69—C70—C71—C72	−0.1 (8)
C12—C13—C14—C15	0.5 (8)	C70—C71—C72—C73	1.0 (8)
C13—C14—C15—C10	1.1 (8)	C71—C72—C73—C68	−0.2 (7)
C11—C10—C15—C14	−2.6 (7)	C69—C68—C73—C72	−1.4 (7)
C2—C10—C15—C14	178.8 (4)	C60—C68—C73—C72	−179.9 (4)
C1—S1—C16—C17	163.3 (3)	C59—S3—C74—C81	−75.1 (3)
C1—S1—C16—C23	−74.8 (3)	C59—S3—C74—C75	162.9 (3)
C23—C16—C17—C22	−31.6 (6)	C81—C74—C75—C76	−30.6 (6)
S1—C16—C17—C22	89.8 (5)	S3—C74—C75—C76	91.1 (5)
C23—C16—C17—C18	152.3 (4)	C81—C74—C75—C80	152.6 (4)
S1—C16—C17—C18	−86.3 (4)	S3—C74—C75—C80	−85.7 (4)
C22—C17—C18—C19	0.3 (7)	C80—C75—C76—C77	1.5 (8)
C16—C17—C18—C19	176.5 (4)	C74—C75—C76—C77	−175.4 (5)
C17—C18—C19—C20	−0.7 (8)	C75—C76—C77—C78	−2.3 (10)
C18—C19—C20—C21	1.6 (9)	C76—C77—C78—C79	2.5 (10)
C19—C20—C21—C22	−2.1 (10)	C77—C78—C79—C80	−2.0 (9)
C20—C21—C22—C17	1.8 (9)	C76—C75—C80—C79	−1.0 (7)
C18—C17—C22—C21	−0.8 (8)	C74—C75—C80—C79	175.9 (4)
C16—C17—C22—C21	−177.0 (5)	C78—C79—C80—C75	1.3 (8)
C17—C16—C23—O1	85.8 (5)	C75—C74—C81—O3	85.2 (5)
S1—C16—C23—O1	−31.8 (5)	S3—C74—C81—O3	−32.9 (5)
C17—C16—C23—C24	−93.8 (4)	C75—C74—C81—C82	−94.6 (4)
S1—C16—C23—C24	148.6 (3)	S3—C74—C81—C82	147.3 (3)
O1—C23—C24—C25	−168.1 (4)	O3—C81—C82—C87	16.5 (6)
C16—C23—C24—C25	11.5 (6)	C74—C81—C82—C87	−163.6 (4)
O1—C23—C24—C29	14.7 (6)	O3—C81—C82—C83	−165.9 (4)
C16—C23—C24—C29	−165.7 (4)	C74—C81—C82—C83	14.0 (6)
C29—C24—C25—C26	0.9 (7)	C87—C82—C83—C84	1.1 (6)
C23—C24—C25—C26	−176.3 (4)	C81—C82—C83—C84	−176.5 (4)
C24—C25—C26—C27	0.3 (8)	C82—C83—C84—C85	1.1 (7)
C25—C26—C27—C28	−0.5 (8)	C83—C84—C85—C86	−2.5 (8)
C25—C26—C27—Cl1	177.1 (4)	C83—C84—C85—Cl3	176.8 (4)
C26—C27—C28—C29	−0.5 (8)	C84—C85—C86—C87	1.6 (7)
Cl1—C27—C28—C29	−178.1 (4)	Cl3—C85—C86—C87	−177.7 (4)
C27—C28—C29—C24	1.7 (7)	C83—C82—C87—C86	−2.0 (6)
C25—C24—C29—C28	−1.9 (7)	C81—C82—C87—C86	175.7 (4)
C23—C24—C29—C28	175.4 (4)	C85—C86—C87—C82	0.7 (7)
C31—N2—C30—C37	−2.3 (6)	C89—N4—C88—C95	−2.7 (6)
C31—N2—C30—S2	175.9 (3)	C89—N4—C88—S4	175.6 (3)
C45—S2—C30—N2	1.8 (4)	C103—S4—C88—N4	1.5 (4)
C45—S2—C30—C37	−179.8 (3)	C103—S4—C88—C95	179.9 (3)
C30—N2—C31—C32	2.0 (6)	C88—N4—C89—C90	2.3 (6)
C30—N2—C31—C39	−179.1 (4)	C88—N4—C89—C97	−178.2 (4)
N2—C31—C32—C38	−0.5 (7)	N4—C89—C90—C96	−0.7 (7)
C39—C31—C32—C38	−179.4 (4)	C97—C89—C90—C96	179.8 (4)
C38—C33—C34—C35	−0.3 (8)	C96—C91—C92—C93	0.0 (8)
C33—C34—C35—C36	0.1 (8)	C91—C92—C93—C94	−0.4 (8)
C34—C35—C36—C37	−0.1 (8)	C92—C93—C94—C95	0.6 (8)
C35—C36—C37—C38	0.3 (7)	C93—C94—C95—C96	−0.4 (7)
C35—C36—C37—C30	179.2 (4)	C93—C94—C95—C88	179.2 (4)
N2—C30—C37—C36	−177.8 (4)	N4—C88—C95—C94	−178.3 (4)
S2—C30—C37—C36	3.9 (6)	S4—C88—C95—C94	3.4 (6)
N2—C30—C37—C38	1.2 (6)	N4—C88—C95—C96	1.4 (6)
S2—C30—C37—C38	−177.1 (3)	S4—C88—C95—C96	−176.9 (3)
C34—C33—C38—C32	−179.3 (5)	C89—C90—C96—C95	−0.6 (7)
C34—C33—C38—C37	0.6 (7)	C89—C90—C96—C91	179.3 (4)
C31—C32—C38—C33	179.2 (4)	C94—C95—C96—C90	180.0 (4)
C31—C32—C38—C37	−0.6 (7)	C88—C95—C96—C90	0.3 (6)
C36—C37—C38—C33	−0.5 (7)	C94—C95—C96—C91	0.1 (6)
C30—C37—C38—C33	−179.5 (4)	C88—C95—C96—C91	−179.6 (4)
C36—C37—C38—C32	179.3 (4)	C92—C91—C96—C90	−179.8 (5)
C30—C37—C38—C32	0.4 (6)	C92—C91—C96—C95	0.1 (7)
C32—C31—C39—C44	155.4 (5)	C90—C89—C97—C102	144.5 (5)
N2—C31—C39—C44	−23.5 (6)	N4—C89—C97—C102	−35.0 (6)
C32—C31—C39—C40	−26.7 (7)	C90—C89—C97—C98	−36.2 (7)
N2—C31—C39—C40	154.4 (4)	N4—C89—C97—C98	144.3 (4)
C44—C39—C40—C41	−2.7 (8)	C102—C97—C98—C99	−1.0 (7)
C31—C39—C40—C41	179.3 (5)	C89—C97—C98—C99	179.7 (4)
C39—C40—C41—C42	1.4 (9)	C97—C98—C99—C100	−0.7 (8)
C40—C41—C42—C43	0.0 (9)	C98—C99—C100—C101	1.8 (8)
C41—C42—C43—C44	0.1 (8)	C99—C100—C101—C102	−1.1 (8)
C40—C39—C44—C43	2.7 (7)	C100—C101—C102—C97	−0.8 (8)
C31—C39—C44—C43	−179.2 (4)	C98—C97—C102—C101	1.8 (7)
C42—C43—C44—C39	−1.5 (8)	C89—C97—C102—C101	−178.9 (5)
C30—S2—C45—C46	−162.6 (3)	C88—S4—C103—C104	−162.9 (3)
C30—S2—C45—C52	75.8 (3)	C88—S4—C103—C110	75.3 (3)
C52—C45—C46—C51	32.9 (6)	C110—C103—C104—C109	33.8 (6)
S2—C45—C46—C51	−88.9 (5)	S4—C103—C104—C109	−87.9 (5)
C52—C45—C46—C47	−150.8 (4)	C110—C103—C104—C105	−150.4 (4)
S2—C45—C46—C47	87.4 (4)	S4—C103—C104—C105	88.0 (4)
C51—C46—C47—C48	−0.8 (7)	C109—C104—C105—C106	−0.5 (7)
C45—C46—C47—C48	−177.2 (4)	C103—C104—C105—C106	−176.6 (4)
C46—C47—C48—C49	0.6 (8)	C104—C105—C106—C107	0.1 (8)
C47—C48—C49—C50	0.1 (9)	C105—C106—C107—C108	0.7 (9)
C48—C49—C50—C51	−0.6 (9)	C106—C107—C108—C109	−1.1 (10)
C49—C50—C51—C46	0.4 (9)	C105—C104—C109—C108	0.2 (8)
C47—C46—C51—C50	0.3 (8)	C103—C104—C109—C108	176.1 (5)
C45—C46—C51—C50	176.6 (5)	C107—C108—C109—C104	0.7 (9)
C46—C45—C52—O2	−87.7 (5)	C104—C103—C110—O4	−87.2 (5)
S2—C45—C52—O2	30.5 (5)	S4—C103—C110—O4	31.0 (5)
C46—C45—C52—C53	92.7 (4)	C104—C103—C110—C111	92.7 (4)
S2—C45—C52—C53	−149.2 (3)	S4—C103—C110—C111	−149.1 (3)
O2—C52—C53—C58	−14.9 (6)	O4—C110—C111—C116	168.7 (4)
C45—C52—C53—C58	164.8 (4)	C103—C110—C111—C116	−11.1 (6)
O2—C52—C53—C54	167.9 (4)	O4—C110—C111—C112	−13.2 (6)
C45—C52—C53—C54	−12.4 (6)	C103—C110—C111—C112	166.9 (4)
C58—C53—C54—C55	−1.7 (7)	C116—C111—C112—C113	2.4 (7)
C52—C53—C54—C55	175.5 (4)	C110—C111—C112—C113	−175.8 (4)
C53—C54—C55—C56	−0.1 (7)	C111—C112—C113—C114	−0.9 (8)
C54—C55—C56—C57	1.8 (7)	C112—C113—C114—C115	−1.0 (8)
C54—C55—C56—Cl2	−177.2 (4)	C112—C113—C114—Cl4	176.5 (4)
C55—C56—C57—C58	−1.6 (7)	C113—C114—C115—C116	1.2 (8)
Cl2—C56—C57—C58	177.4 (4)	Cl4—C114—C115—C116	−176.3 (4)
C54—C53—C58—C57	1.9 (7)	C114—C115—C116—C111	0.4 (8)
C52—C53—C58—C57	−175.4 (4)	C112—C111—C116—C115	−2.2 (7)
C56—C57—C58—C53	−0.3 (7)	C110—C111—C116—C115	175.9 (4)
